# Bilateral disease in the classic subtype of papillary thyroid carcinoma: clinical significance and development of an artificial intelligence-based multimodal prediction model

**DOI:** 10.3389/fendo.2026.1759451

**Published:** 2026-05-20

**Authors:** Wan-Xiao Wu, Yu-Xin Yang, Jia-Wei Feng, Shui-Qing Liu, An-Cheng Qin, Yong Jiang

**Affiliations:** 1 Department of Thyroid Surgery, The Third Affiliated Hospital of Soochow University, Changzhou First People’s Hospital, Changzhou, Jiangsu, China; 2 Department of Ultrasound, The Third Affiliated Hospital of Soochow University, Changzhou First People’s Hospital, Changzhou, Jiangsu, China; 3 Department of Thyroid Surgery, Suzhou Municipal Hospital, The Affiliated Suzhou Hospital of Nanjing Medical University, Suzhou, Jiangsu, China

**Keywords:** artificial intelligence, bilateral disease, multifocality, multimodal model, papillary thyroid carcinoma

## Abstract

**Background:**

Papillary thyroid carcinoma (PTC) is the most common thyroid malignancy, with multifocality being a frequent occurrence. The clinical significance of bilateral disease versus unilateral multifocality remains controversial. This study aimed to evaluate the clinicopathological characteristics and recurrence patterns of bilateral disease in patients with the classic subtype of PTC, and to develop a multimodal artificial intelligence model for preoperative identification of bilateral disease in this specific histological subtype.

**Methods:**

Clinical data and ultrasound images of 1,218 patients with PTC were retrospectively collected from two hospitals. Clinicopathological features and recurrence patterns were analyzed. Four prediction models were developed: a logistic regression model with clinical parameters (Model A), a machine learning-based model with LASSO-selected features (Model B), a deep learning framework using TresNet architecture (Model C), and an integrated model combining Models B and C (Model D). Model performance was assessed in internal and external validation cohorts, with clinical utility evaluated across different radiologist experience levels.

**Results:**

Among the 1,098 patients from the primary institution, 26.1% had multifocal disease, of which 62.7% had bilateral involvement. Bilateral disease was associated with larger tumor size, higher rates of lymph node metastasis, and worse recurrence-free survival compared to unifocal disease and unilateral multifocality. Multivariate analysis identified bilateral disease as an independent risk factor for recurrence (HR = 9.664, *P* = 0.005). The integrated Model D achieved superior performance with AUC of 0.970 in the training set, 0.932 in the validation set, and 0.848 in the external validation set. In the external validation group of 120 patients, Model D’s implementation significantly improved diagnostic accuracy across all radiologist experience levels, with junior radiologists benefiting most substantially (+20.9% improvement).

**Conclusion:**

In classic subtype PTC, bilateral disease, rather than multifocality alone, is a significant risk factor for recurrence. The developed multimodal artificial intelligence model effectively predicts bilateral disease preoperatively, potentially guiding surgical planning and improving diagnostic accuracy irrespective of radiologist experience.

## Background

Papillary thyroid carcinoma (PTC) accounts for 80-90% of thyroid malignancies (1). Its incidence has risen significantly worldwide due to advanced imaging technologies. Multifocality, defined as two or more tumor foci within the thyroid gland, occurs in 20-36.1% of PTC cases, with bilateral involvement representing a significant subset of these cases (2, 3).

The clinical significance of multifocality and bilaterality in PTC remains controversial. Some studies have demonstrated that multifocality, particularly bilateral disease, is associated with aggressive clinicopathological features, increased risk of lymph node metastasis, and higher recurrence rates (2, 4–7). However, others have reported that patients with multifocal PTC have similar outcomes to those with unifocal disease (8, 9). The 8th edition of the American Joint Committee on Cancer staging system does not incorporate multifocality as a prognostic factor, reflecting this ongoing debate (10).

Preoperative identification of bilateral disease is crucial for surgical planning. However, a considerable proportion of bilateral cases are only discovered incidentally during postoperative histopathological examination. Research indicates that approximately 11.9-15.5% of bilateral tumors are not detected preoperatively despite modern imaging techniques (11, 12).

Recent advances in artificial intelligence and radiomics show potential for improving diagnostic accuracy in thyroid cancer (13, 14). Radiomics extracts quantitative features from medical images that capture subtle tissue characteristics beyond visual assessment (15, 16). Additionally, various systemic inflammatory markers have emerged as potential biomarkers for predicting tumor aggressiveness and prognosis in various cancers, including PTC (17, 18). However, few studies have comprehensively investigated the integration of clinical parameters, inflammatory indices, and radiomics features for predicting bilateral disease in PTC.

This study aims to evaluate the clinicopathological characteristics and recurrence risk patterns of unifocal, unilateral multifocal, and bilateral disease in patients with the classic subtype of PTC, identify independent predictors of bilateral disease, develop and validate a multimodal artificial intelligence model integrating clinical parameters, inflammatory indices, radiomics features, and ultrasound images for preoperative identification of bilateral disease, and assess the clinical utility of this model in enhancing diagnostic accuracy across different levels of radiologist experience.

## Materials and methods

### Patients selection

Clinical data and ultrasound images of 1,218 patients with PTC were retrospectively collected from January 2022 to July 2023 from two hospitals in China: Changzhou First People’s Hospital (n=1,098) and Suzhou Municipal Hospital (n=120). The study protocol was approved by the Institutional Review Boards of Changzhou First People’s Hospital and Suzhou Municipal Hospital. All procedures were performed in accordance with the ethical standards of the 2013 Declaration of Helsinki. Inclusion criteria comprised: (1) histologically confirmed classic (conventional) subtype of PTC according to the 2022 WHO Classification of Endocrine and Neuroendocrine Tumors, (2) availability of high-resolution preoperative ultrasound images, (3) complete clinical and follow-up data, (4) no prior thyroid interventions, and (5) patients aged 18 or older. Exclusion criteria were: PTC of non-classic histological subtypes, including aggressive variants known to be associated with more adverse biological behavior and less favorable prognosis (tall cell, hobnail, columnar cell, solid/trabecular, and diffuse sclerosing variants), as well as other recognized non-classic variants (follicular variant, encapsulated variant, and cribriform-morular variant); other thyroid cancers (e.g., medullary, follicular, anaplastic, or poorly differentiated carcinoma); prior thyroid treatment; concomitant malignancies; inadequate imaging quality; incomplete records; loss to follow-up; or evidence of residual disease within six months post-intervention.

### Definitions of key parameters

Anthropometric measurements were obtained at initial presentation. Body mass index (BMI) was calculated as weight in kilograms divided by height in meters squared (kg/m²) and categorized according to World Health Organization classification. Chronic lymphocytic thyroiditis (CLT) was diagnosed based on elevated anti-thyroid peroxidase antibody levels or characteristic sonographic patterns of diffuse parenchymal heterogeneity. Extrathyroidal extension was defined ultrasonographically as tumor-capsule interface exceeding 25% of the tumor circumference.

For the purposes of this investigation, multifocality was defined as the histopathological identification of two or more foci of PTC within the resected thyroid tissue. Bilateral disease was characterized by histological evidence of PTC in both thyroid lobes. Ipsilateral multifocality referred to patients with multiple PTC foci in the ipsilateral thyroid lobe, without the presence of contralateral cancers. For cases involving the isthmus, classification was based on the anatomical distribution of tumor foci as confirmed by postoperative histopathological mapping: (1) patients with tumor foci confined to one thyroid lobe and/or the isthmus, without any focus in the contralateral lobe, were classified as having unilateral disease (either unifocal or unilateral multifocal, depending on the number of foci); (2) patients with at least one tumor focus in each of the two thyroid lobes were classified as having bilateral disease, irrespective of whether an additional isthmic focus was present; and (3) an isolated isthmic tumor without lobar involvement was classified as unilateral unifocal disease. Contralateral occult PTC was defined as PTC foci in the contralateral thyroid lobe not detected preoperatively by ultrasound or fine-needle aspiration, but identified only during histopathological examination of surgical specimens. Importantly, the inclusion criteria required every tumor focus in each case to be of the classic histological subtype. Therefore, any patient in whom one or more additional foci demonstrated a non-classic variant — including aggressive variants (tall cell, hobnail, columnar cell, solid/trabecular, and diffuse sclerosing) as well as other non-classic variants (follicular, encapsulated, and cribriform-morular) — was fully excluded from the cohort and was not retained by reclassifying the case according to the histological subtype of the largest focus. Consequently, all multifocal cases included in this study were histologically homogeneous, with every focus representing classic PTC. On this basis, quantitative pathological parameters of the index lesion (including maximum tumor diameter, A/T ratio, margin characteristics, extrathyroidal extension status, and lymphovascular involvement) were reported based on the largest tumor focus, because histological subtype was uniform across foci by study design.

Disease recurrence was defined as the detection of new tumor manifestations occurring at least 12 months after initial therapeutic intervention, thus distinguishing it from persistent disease. Recurrence patterns were classified as locoregional (involving the thyroid bed or cervical lymph nodes) or distant (affecting non-regional sites). Recurrence was established through: (1) cytological confirmation, or (2) biochemical evidence of disease activity with corresponding structural abnormalities.

### Ultrasonographic assessment

Ultrasound examinations were performed using standardized protocols on high-resolution imaging systems (Philips iU22, GE LOGIQ E9, Siemens ACUSON, or Toshiba Aplio) by sonographers with a minimum of 10 years of thyroid imaging experience. For each case, axial grayscale images capturing the maximum dimension of the primary lesion were archived in DICOM format for subsequent analysis.

### Image processing and feature extraction

Raw ultrasound images underwent standardized preprocessing using a uniform pipeline. Images were resampled to isotropic voxel dimensions (1×1×1 mm³) and standardized with 25 grayscale intensity discretization bins using 3D-Slicer software (version 4.10.2). The cross-sectional plane demonstrating maximum tumor diameter was selected, with grayscale intensities normalized to a [-1, 1] range. Regions of interest (ROIs) were extracted and standardized to 224×224 pixels via nearest-neighbor interpolation algorithms. ROI delineation was performed manually using 3D-Slicer by two board-certified radiologists with extensive experience in thyroid imaging. Primary segmentation was conducted by a senior radiologist (>10 years of experience) who was blinded to clinical outcomes. A second radiologist (>5 years of experience) independently performed segmentation for assessment of inter-observer variability. Reproducibility was evaluated through intraclass correlation coefficient analysis, yielding values of 0.846-0.943 for intra-observer and 0.891-0.940 for inter-observer comparisons, indicating robust measurement reliability.

### Radiomics analysis and predictive modeling

Radiomic features were extracted in accordance with the Image Biomarker Standardization Initiative guidelines using the pyradiomics library (version 3.0.0). A comprehensive set of 846 quantitative imaging features was derived from segmented ultrasound ROIs, including morphological, first-order statistical, texture, and wavelet transform features. Further refinement through mutual information assessment and statistical filtering isolated a subset of highly relevant ultrasound-derived signatures, which were subsequently integrated with pertinent preoperative clinical parameters to create a composite feature dataset. A rigorous 10-fold cross-validation protocol was implemented with Least Absolute Shrinkage and Selection Operator (LASSO) regression to identify an optimal combination of clinical and radiomic predictive variables for model construction.

Four complementary prediction models were developed: (1) Model A: A logistic regression model incorporating preoperative and postoperative clinical parameters. (2) Model B: A machine learning-based approach utilizing LASSO-selected features. Multiple algorithms (Random Forest, Gradient Boosting Machine, Support Vector Machine, XGBoost, and K-Nearest Neighbors) were systematically evaluated. Support Vector Machine (SVM) demonstrated superior performance and was subsequently optimized using 10-fold cross-validation with the scikit-learn package (version 1.6.0). (3) Model C: A deep learning framework based on convolutional neural networks for image-based prediction. Transfer learning with pre-trained ImageNet weights was implemented to enhance generalization. Various architectures (TresNet, ResNet-34, ResNet-101, ResNet-18, and Inception-3) were comprehensively evaluated, with TresNet emerging as the optimal backbone. The final model employed cross-entropy loss function and Adam optimizer with learning rate decay strategy. Image augmentation techniques including random cropping, flipping, and rotational transformations were applied to mitigate overfitting. (4) Model D: An integrated approach combining probabilistic outputs from the machine learning and deep learning models (models B and C) through calibrated logistic regression coefficients.

### Model validation and clinical implementation

Model performance was initially assessed using the internal validation cohort and subsequently confirmed through external validation. To evaluate real-world clinical utility, three cohorts of radiologists stratified by experience level (junior: 1–5 years, intermediate: 5–10 years, senior: >10 years) evaluated cases from the external validation set before and after implementation of the integrated prediction system. Diagnostic accuracy, sensitivity, specificity, and confidence ratings were systematically documented and compared.

### Interpretability analysis

To enhance model transparency, Gradient-weighted Class Activation Mapping (Grad-CAM) was applied to generate heat maps highlighting influential regions in classification decisions. SHapley Additive exPlanations (SHAP) analysis quantified feature contributions to model predictions, using TreeExplainer to calculate SHAP values for all features in the integrated Model D. This provided both global feature importance rankings and local case-specific interpretations. Sankey diagrams illustrated diagnostic information flow across prediction models and radiologist experience levels.

### Statistical analysis

Statistical analyses were performed using SPSS (version 25.0), R (version 4.1.2), and Python (version 3.9.0). Categorical variables were compared using Pearson’s chi-square test or Fisher’s exact test. Continuous variables were analyzed with Student’s t-test or Mann-Whitney U test based on distribution characteristics. Model discrimination was assessed through receiver operating characteristic (ROC) curve analysis with area under the curve (AUC) calculation. Comparisons between ROC curves were performed using DeLong’s test. Comprehensive performance metrics including accuracy, sensitivity, specificity, positive predictive value (PPV), negative predictive value (NPV), and F1 score were calculated for each model. Multivariate logistic regression identified independent predictors of bilateral disease, following initial univariate screening (P ≤ 0.05). Results were expressed as odds ratios (ORs) with 95% confidence intervals (CIs). The final model incorporated pre-determined risk factors while excluding variables with demonstrated collinearity. Recurrence-free survival (RFS) was calculated using the Kaplan-Meier method, with differences between groups assessed by the log-rank test. Cox proportional hazards modeling identified independent predictors of recurrence, with results expressed as hazard ratios (HRs) and 95% confidence intervals. Two-sided *P*-values <0.05 were considered statistically significant for all analyses.

## Results

### Clinicopathological features of multifocality and bilaterality

Among 1,098 patients with classic PTC form Changzhou First People’s Hospital, 811 patients (73.9%) presented with unifocal disease (UFD), while 287 patients (26.1%) had multifocal disease (MFD). Within the MFD group, 107 patients (37.3%) had unilateral MFD and 180 patients (62.7%) had bilateral disease (**Supplementary Figure 1**). The clinicopathological characteristics of these groups are presented in **Table 1**.

**Table 1:** Clinicopathologic features of multifocality and bilaterality.

Characteristics	Total	UFD	MFD		*P* value^*^	*P* value^+^	*P* value^#^
			Unilateral MFD	Bilateral disease			
	(N = 1098)	(N = 811)	(N = 107)	(N = 180)			
Sex (Female)	825 (75.1%)	608 (75.0%)	79 (73.8%)	138 (76.7%)	0.798	0.632	0.589
Age, years	43.57 ± 11.62	43.33 ± 11.71	42.86 ± 11.00	45.06 ± 11.45	0.679	0.053	0.104
<55	909 (82.8%)	674 (83.1%)	91 (85.0%)	144 (80.0%)	0.621	0.311	0.271
BMI, kg/m^2^	24.13 ± 3.70	23.95 ± 3.70	24.79 ± 4.04	24.57 ± 3.42	0.024	0.039	0.613
BRAF V600E mutation (Positive)	982 (89.4%)	717 (88.4%)	97 (90.7%)	168 (93.3%)	0.485	0.042	0.404
CLT (Presence)	254 (23.1%)	173 (21.3%)	27 (25.2%)	54 (30.0%)	0.350	0.011	0.372
Tumor size, cm	1.05 ± 0.72	1.03 ± 0.72	0.95 ± 0.54	1.24 ± 0.79	0.251	<0.001	<0.001
≤1	731 (66.6%)	563 (69.4%)	74 (69.2%)	94 (52.5%)			
>1 to ≤2	274 (25.0%)	180 (22.2%)	28 (26.2%)	66 (36.7%)			
>2 to ≤4	84 (7.7%)	61 (7.5%)	5 (4.7%)	18 (10.0%)			
>4	9 (0.8%)	7 (0.9%)	0 (0.0%)	2 (1.1%)	0.335	<0.001	0.033
The number of foci							
1	811 (73.9%)	811 (100.0%)	0 (0.0%)	0 (0.0%)			
2	213 (19.4%)	0 (0.0%)	95 (88.8%)	118 (65.6%)			
3 or more	74 (6.7%)	0 (0.0%)	12 (11.2%)	62 (34.4%)	<0.001	<0.001	<0.001
Location							
Upper	238 (21.7%)	191 (23.6%)	19 (17.8%)	28 (15.6%)			
Isthmus	65 (5.9%)	31 (3.8%)	4 (3.7%)	30 (16.7%)	0.048	<0.001	0.004
Margin							
Lobulated/irregular	275 (25.0%)	211 (26.0%)	23 (21.5%)	41 (22.8%)			
ETE	36 (3.3%)	27 (3.3%)	1 (0.9%)	8 (4.4%)	0.555	0.845	0.740
A/T (≥ 1)	764 (69.6%)	541 (66.7%)	76 (71.0%)	147 (81.7%)	0.375	<0.001	0.026
US-reported CLN status (Suspected)	171 (15.6%)	115 (14.2%)	14 (13.1%)	42 (23.3%)	0.741	0.003	0.032
CLNM (Positive)	331 (30.1%)	214 (26.4%)	35 (32.7%)	82 (45.6%)	0.158	<0.001	0.025
High-volume CLNM (Positive)	126 (11.5%)	75 (9.2%)	12 (11.2%)	39 (21.7%)	0.499	<0.001	0.022
CLNR	0.19 ± 0.28	0.18 ± 0.28	0.22 ± 0.28	0.22 ± 0.26	0.153	0.078	0.964
>0.5	185 (16.8%)	135 (16.6%)	19 (17.8%)	31 (17.2%)	0.768	0.853	0.904
LLNM (Positive)	86 (7.8%)	47 (5.8%)	10 (9.3%)	29 (16.1%)	0.146	<0.001	0.093
High-volume LLNM (Positive)	46 (4.2%)	23 (2.8%)	6 (5.6%)	17 (9.4%)	0.146	<0.001	0.093
No. of removed LNs in CC	8.42 ± 5.22	7.48 ± 4.33	8.14 ± 5.58	12.82 ± 6.34	0.152	<0.001	<0.001
No. of removed LNs in LC	2.62 ± 8.64	1.98 ± 7.61	3.40 ± 9.97	5.04 ± 11.28	0.063	<0.001	0.185
No. of metastatic LNs in CC	1.52 ± 2.49	1.23 ± 2.00	1.70 ± 2.63	2.74 ± 3.72	0.025	<0.001	0.019
No. of metastatic LNs in LC	0.45 ± 1.74	0.32 ± 1.52	0.65 ± 2.21	0.89 ± 2.22	0.052	<0.001	0.413
Size of metastatic LNs in CC, cm	0.75 ± 0.39	0.72 ± 0.37	0.75 ± 0.41	0.87 ± 0.43	0.441	<0.001	0.019
Size of metastatic LNs in LC, cm	1.83 ± 0.60	1.81 ± 0.60	1.86 ± 0.54	1.85 ± 0.62	0.651	0.584	0.919
TG, ng/ml	32.98 ± 78.28	33.54 ± 77.96	27.91 ± 54.50	33.46 ± 90.63	0.461	0.992	0.577
TG-Ab, IU/ml	119.23 ± 424.92	110.45 ± 415.19	83.80 ± 243.89	179.87 ± 533.48	0.424	0.042	0.038
TPO-Ab, IU/ml	37.26 ± 84.06	37.91 ± 86.22	26.48 ± 70.26	40.72 ± 81.16	0.170	0.685	0.138
LMR	5.75 ± 4.60	5.92 ± 4.71	5.79 ± 4.45	4.97 ± 4.07	0.591	< 0.001	0.041
NLR	4.15 ± 2.70	4.00 ± 2.67	4.41 ± 2.76	4.67 ± 2.71	0.125	0.002	0.409
PLR	237.12 ± 146.72	229.38 ± 144.74	248.83 ± 157.42	265.03 ± 145.08	0.204	0.003	0.354
SII	1427.73 ± 1176.12	1358.27 ± 1157.38	1565.19 ± 1227.41	1658.98 ± 1191.84	0.091	0.004	0.512
Risk stratification							
Intermediate	412 (37.5%)	303 (37.4%)	34 (31.8%)	75 (41.7%)			
High	10 (0.9%)	7 (0.9%)	0 (0.0%)	3 (1.7%)	0.266	0.002	0.049
Recurrence (Presence)	23 (2.09%)	14 (1.73%)	3 (2.80%)	6 (3.33%)	0.483	0.149	0.821

UFD, unifocal disease; MFD, multifocal disease; SD, standard deviation; BMI, body mass index; CLT, chronic lymphocytic thyroiditis; A/T, Anteroposterior to Transverse ratio; ETE, extrathyroidal extension; US, Ultrasound; CLN, central lymph node; CLNM, central lymph node metastasis; LLNM, lateral lymph node metastasis; LN, lymph node; CC, central compartment; LC, lateral compartment; TG, Thyroglobulin; TG-Ab, anti-thyroglobulin antibodies; TPO-Ab, thyroid peroxidase antibody; LMR, Lymphocyte to monocyte ratio; NLR, Neutrophil to lymphocyte ratio; PLR, Platelet to lymphocyte ratio; SII, Systemic Immune-inflammation Index.

*P* value^*^: UFD vs. Unilateral MFD; *P* value^+^: UFD vs. Bilateral disease; *P* value^#^: Unilateral MFD vs. Bilateral disease.

Compared to patients with UFD, those with bilateral disease showed significantly higher BMI (24.57 ± 3.42 vs. 23.95 ± 3.70 kg/m², *P* = 0.039), higher BRAF V600E mutation prevalence (93.3% vs. 88.4%, *P* = 0.042), and increased CLT rates (30.0% vs. 21.3%, *P* = 0.011). Bilateral disease was associated with larger tumor size (1.24 ± 0.79 vs. 1.03 ± 0.72 cm, *P* < 0.001), higher frequency of isthmus location (16.7% vs. 3.8%, *P* < 0.001), and significantly higher rates of aspect to transverse (A/T) ratio ≥1 (81.7% vs. 66.7%, *P* < 0.001), central lymph node metastasis (CLNM) (45.6% vs. 26.4%, *P* < 0.001), and lateral lymph node metastasis (16.1% vs. 5.8%, *P* < 0.001). When compared with unilateral MFD patients, bilateral disease was characterized by larger tumor size (1.24 ± 0.79 vs. 0.95 ± 0.54 cm, *P* < 0.001), higher frequency of isthmus location (16.7% vs. 3.7%, *P* = 0.004), and greater proportion of cases with 3 or more foci (34.4% vs. 11.2%, *P* < 0.001). Additionally, bilateral disease showed higher rates of CLNM (45.6% vs. 32.7%, *P* = 0.025) and greater size of metastatic LNs in central compartment (0.87 ± 0.43 vs. 0.75 ± 0.41 cm, *P* = 0.019).

### Recurrence risk factors

**Figure 1:**
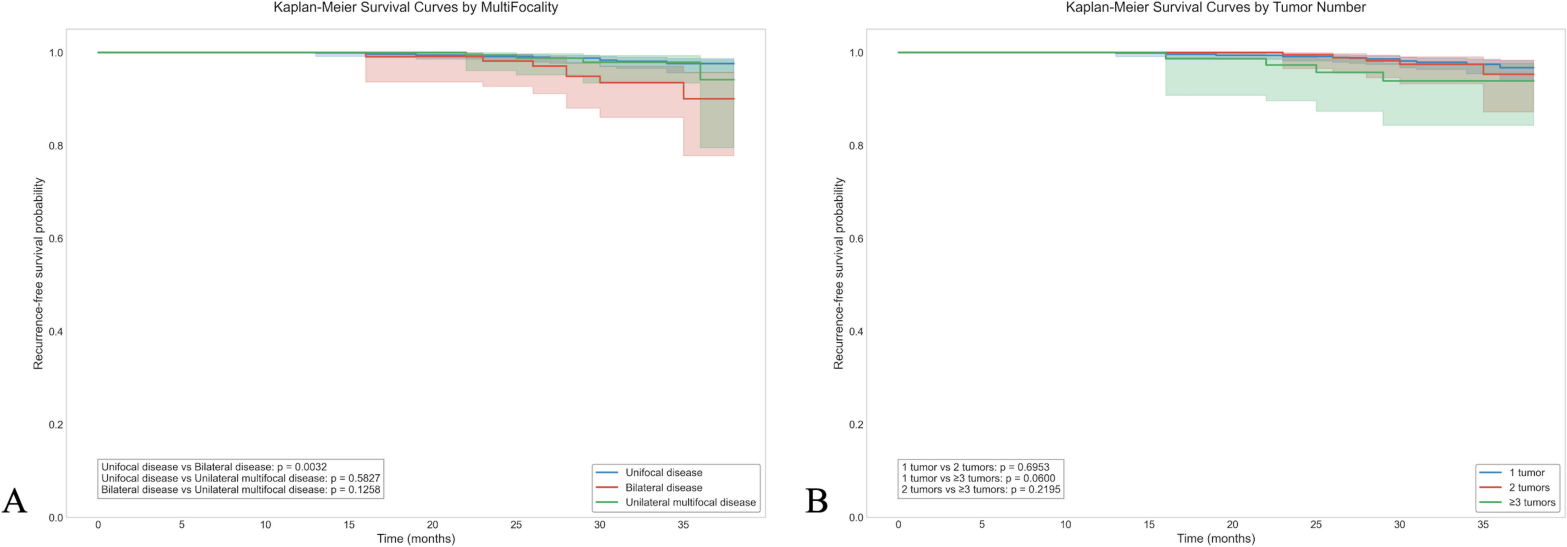
Recurrence-free survival analysis of PTC patients. **(A)** Kaplan-Meier survival curves stratified by multifocality pattern demonstrating significantly worse recurrence-free survival in patients with bilateral disease compared to those with unifocal disease (*P* = 0.0032), while no significant difference was observed between unifocal disease and unilateral multifocal disease (p=0.5827). **(B)** Kaplan-Meier survival curves stratified by tumor number showing no significant differences in recurrence-free survival between groups with 1, 2, or ≥3 tumor foci (all *P*>0.05).

During a median follow-up of 29.9 months (range: 19–38 months), 23 patients (2.09%) experienced recurrence. The Kaplan-Meier survival curves (**Figure 1A**) demonstrated that patients with bilateral disease had significantly worse recurrence-free survival compared to those with UFD (*P* = 0.0032), while no significant difference was observed between UFD and unilateral MFD (*P* = 0.5827). **Figure 1B** shows that the number of tumor foci did not significantly impact recurrence-free survival (all *P >*0.05).

In multivariate Cox regression analysis (**Table 2**), bilateral disease emerged as an independent risk factor for recurrence (HR = 9.664, 95% CI: 1.955-47.775, *P* = 0.005), along with central lymph node ratio >0.5 (HR = 6.319, 95% CI: 1.052-37.965, *P* = 0.044), size of metastatic LNs in lateral compartment (HR = 4.959, 95% CI: 1.041-23.623, *P* = 0.044), high NLR (HR = 2.689, 95% CI: 1.262-5.729, *P* = 0.010), and high-risk stratification (HR = 14.156, 95% CI: 1.012-198.067, *P* = 0.049).

**Table 2:** Multivariate analyses of recurrence risk factors.

Characteristics	Multivariate		
	HR	(95% CI)	*P* value
Sex (Female)	2.147	0.136-33.955	0.588
Age (≥55)	0.951	0.873-1.035	0.247
A/T (≥ 1)	0.357	0.055-2.309	0.279
BRAF V600E mutation (Positive)	0.356	0.086-1.483	0.156
CLT (Presence)	0.270	0.051-1.438	0.125
Tumor size, cm	1.376	0.62-2.291	0.288
Multifocality			
Unilateral MFD	0.982	0.229-4.206	0.981
Bilateral disease	9.664	1.955-47.775	0.005
The number of foci			
2	0.223	0.002-21.021	0.518
3 or more	2.552	0.098-66.560	0.573
Location			
Middle/Lower	0.155	0.019-1.281	0.084
Isthmus	2.164	0.226-20.708	0.503
Margin			
Lobulated or irregular	8.759	0.845-90.765	0.069
ETE	0.338	0.011-10.618	0.538
CLNM (Positive)	0.162	0.008-3.194	0.231
High-volume CLNM (Positive)	2.614	0.436-15.661	0.293
CLNR (>0.5)	6.319	1.052-37.965	0.044
LLNM (Positive)	6.651	0.328-134.729	0.217
High-volume LLNM (Positive)	3.494	0.835-14.617	0.087
Size of metastatic LNs in CC, cm	4.826	0.465-50.057	0.187
Size of metastatic LNs in LC, cm	4.959	1.041-23.623	0.044
Serum TG, ng/ml	0.995	0.984-1.006	0.374
Serum TG-Ab, IU/ml	1.001	1.000-1.002	0.098
Serum TPO-Ab, IU/ml	0.994	0.980-1.008	0.367
LMR	0.873	0.572-1.334	0.530
NLR	2.689	1.262-5.729	0.035
PLR	1.008	0.996-1.019	0.205
SII	0.999	0.993-1.004	0.638
Risk stratification			
Intermediate	5.056	0.496-51.508	0.171
High	14.156	1.012-198.067	0.049

UFD, unifocal disease; MFD, multifocal disease; BMI, body mass index; CLT, chronic lymphocytic thyroiditis; A/T, Anteroposterior to Transverse ratio; ETE, extrathyroidal extension; US, Ultrasound; CLN, central lymph node; CLNM, central lymph node metastasis; LLNM, lateral lymph node metastasis; LN, lymph node; CC, central compartment; LC, lateral compartment; TG, Thyroglobulin; TG-Ab, anti-thyroglobulin antibodies; TPO-Ab, thyroid peroxidase antibody; LMR, Lymphocyte to monocyte ratio; NLR, Neutrophil to lymphocyte ratio; PLR, Platelet to lymphocyte ratio; SII, Systemic Immune-inflammation Index; HR, hazard ratio; CI, confidence interval.

### Predictive factors for bilateral disease

Among the 180 patients with bilateral disease, 32 (17.8%) had contralateral occult PTC (**Supplementary Table 1**) with mean tumor size of 0.71 ± 0.44 cm. Only 18.8% of these cases underwent preoperative fine-needle aspiration, all yielding negative results, with definitive diagnosis established only through intraoperative frozen section or postoperative pathology. For model development, we divided the 1,098 patients into training and validation sets. The training set comprised 148 non-incidentally discovered bilateral cases and 755 stratified-sampled unilateral cases, while the validation set included 32 incidentally discovered bilateral cases and 163 unilateral cases, maintaining an approximately 82.2%:17.8% ratio to ensure balanced clinical feature distribution between cohorts (**Supplementary Figure 1**; **Table 3**).

**Table 3:** Demographics of patients from the training, validation and external validation sets.

Characteristics	Training set	Validation set	External validation set	*P* value*
	(N = 903)	(N = 195)	(N = 120)	
Bilateral disease	148 (16.4%)	32 (16.4%)	23 (19.2%)	0.994
Multifocal disease	233 (25.8%)	54 (27.7%)	33 (27.5%)	0.659
Sex (Female)	228 (25.2%)	45 (23.1%)	37 (30.8%)	0.524
Age (<55 years)	742 (82.2%)	167 (85.6%)	101 (84.2%)	0.244
BMI, kg/m^2^	24.03 ± 3.78	24.61 ± 3.32	24.22 ± 3.91	0.057
BRAF V600E mutation (Positive)	806 (89.3%)	176 (90.3%)	109 (90.8%)	0.681
CLT (Presence)	214 (23.7%)	40 (20.5%)	30 (25.0%)	0.339
Tumor size, cm	1.06 ± 0.73	1.01 ± 0.67	1.07 ± 0.70	0.376
Margin				
Lobulated or irregular	222 (24.6%)	53 (27.1%)	41 (34.2%)	
ETE	34 (3.8%)	2 (1.0%)	4 (3.3%)	0.074
A/T (≥ 1)	617 (68.3%)	147 (75.4%)	82 (68.3%)	0.052
CLNM (Positive)	273 (30.2%)	58 (29.7%)	59 (49.2%)	0.893
High-volume CLNM (Positive)	103 (11.4%)	23 (11.8%)	21 (17.5%)	0.877
CLNR	0.19 ± 0.28	0.20 ± 0.28	0.21 ± 0.25	0.421
LLNM (Positive)	71 (7.9%)	15 (7.7%)	17 (14.2%)	0.936
High-volume LLNM (Positive)	39 (4.3%)	7 (3.6%)	6 (5.0%)	0.645
Size of metastatic LNs in CC, cm	0.75 ± 0.39	0.74 ± 0.38	0.76 ± 0.33	0.847
Size of metastatic LNs in LC, cm	1.82 ± 0.57	1.96 ± 0.59	1.88 ± 0.61	0.377
Serum TG, ng/ml	33.53 ± 79.61	30.43 ± 72.19	31.58 ± 65.19	0.501
Serum TG-Ab, IU/ml	129.74 ± 463.26	70.59 ± 145.62	53.28 ± 105.70	0.078
Serum TPO-Ab, IU/ml	39.36 ± 88.71	27.53 ± 57.31	146.70 ± 541.85	0.075
LMR	5.82 ± 4.49	5.43 ± 5.07	5.67 ± 4.82	0.282
NLR	4.34 ± 2.69	4.66 ± 2.69	4.61 ± 2.83	0.051
PLR	250.34 ± 145.65	268.51 ± 148.65	271.13 ± 147.39	0.066
SII	1377.88 ± 1171.12	1658.57 ± 1177.68	1478.81 ± 1182.91	0.071
Risk stratification				
Intermediate	332 (36.8%)	80 (41.0%)	36 (30.0%)	
High	8 (0.9%)	2 (1.0%)	2 (1.7%)	0.276

BMI, body mass index; CLT, chronic lymphocytic thyroiditis; A/T Anteroposterior to Transverse ratio; ETE, extrathyroidal extension; CLN, central lymph node; CLNM, central lymph node metastasis; LLNM, lateral lymph node metastasis; LN, lymph node; CC, central compartment; LC, lateral compartment; TG, Thyroglobulin; TG-Ab, anti-thyroglobulin antibodies; TPO-Ab, thyroid peroxidase antibody; LMR, Lymphocyte to monocyte ratio; NLR, Neutrophil to lymphocyte ratio; PLR, Platelet to lymphocyte ratio; SII, Systemic Immune-inflammation Index.

*The *P*-value reflects the comparison between the training and validation sets.

Multivariate logistic regression analysis (**Table 4**) identified BRAF V600E mutation (OR = 9.243, 95% CI: 1.069-79.905, *P* = 0.043), A/T ratio ≥1 (OR = 5.080, 95% CI: 1.700-15.181, *P* = 0.004), high-volume CLNM (OR = 5.729, 95% CI: 1.659-19.780, *P* = 0.006), and size of metastatic LNs in central compartment (OR = 2.766, 95% CI: 1.056-7.245, *P* = 0.038) as independent predictors of bilateral disease.

**Table 4:** Logistic regression odds model for bilateral disease (Model A).

Characteristics	Multivariate		
	OR	(95% CI)	*P* value
Sex (Female)	0.273	0.058-1.287	0.101
Age (<55)	5.814	0.722-46.831	0.098
BMI, kg/m^2^	1.068	0.897-1.270	0.461
BRAF V600E mutation (Positive)	9.243	1.069-79.905	0.043
CLT (Presence)	0.462	0.090-2.360	0.353
Tumor size, cm	0.655	0.378-1.136	0.132
Margin			
Lobulated or irregular	2.259	0.578-8.823	0.241
ETE	0.536	0.065-4.447	0.564
A/T (≥ 1)	5.080	1.700-15.181	0.004
US-reported CLN status (Suspected)	0.557	0.026-11.709	0.706
CLNM (Positive)	4.096	0.392-42.835	0.239
High-volume CLNM (Positive)	5.729	1.659-19.780	0.006
CLNR	0.121	0.015-0.959	0.060
LLNM (Positive)	2.537	0.529-10.884	0.210
High-volume LLNM (Positive)	1.640	0.507-5.304	0.409
Size of metastatic LNs in CC, cm	2.766	1.056-7.245	0.038
Size of metastatic LNs in LC, cm	0.305	0.083-1.124	0.074
Serum TG, ng/ml	1.000	0.996-1.005	0.857
Serum TG-Ab, IU/ml	1.000	1.000-1.001	0.359
Serum TPO-Ab, IU/ml	0.999	0.994-1.005	0.761
LMR	0.936	0.692-1.265	0.666
NLR	0.915	0.539-1.553	0.743
PLR	0.996	0.984-1.008	0.543
SII	1.000	0.996-1.003	0.944
Risk stratification			
Intermediate	0.233	0.006-9.094	0.436
High	0.011	0.000-3.350	0.123

BMI, body mass index; CLT, chronic lymphocytic thyroiditis; ETE, extrathyroidal extension; A/T, Anteroposterior to Transverse ratio; US, Ultrasound; CLN, central lymph node; CLNM, central lymph node metastasis; LLNM, lateral lymph node metastasis; LN, lymph node; CC, central compartment; LC, lateral compartment; TG, Thyroglobulin; TG-Ab, anti-thyroglobulin antibodies; TPO-Ab, thyroid peroxidase antibody; LMR, Lymphocyte to monocyte ratio; NLR, Neutrophil to lymphocyte ratio; PLR, Platelet to lymphocyte ratio; SII, Systemic Immune-inflammation Index; OR, odds ratio; CI, confidence interval.

**Figure 2:**
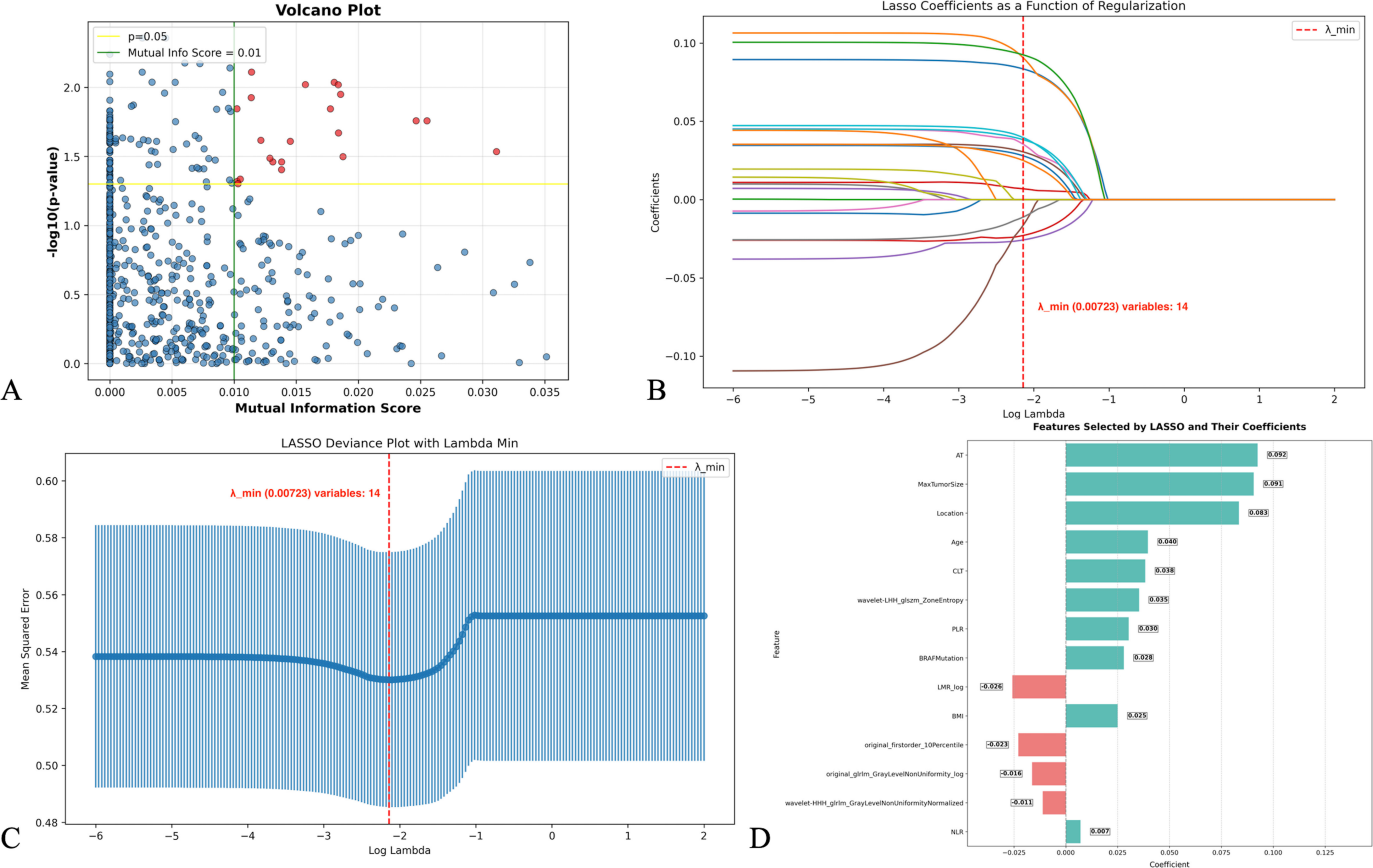
Radiomics feature selection process. **(A)** Volcano plot displaying differential expression of radiomics features based on mutual information score (x-axis) and statistical significance (y-axis) with thresholds set at p=0.05 and mutual information score=0.01. **(B)** LASSO coefficient trajectories as a function of the regularization parameter (log lambda), with the vertical dashed line indicating λ_min (0.00723). **(C)** LASSO deviance plot showing mean squared error with corresponding confidence intervals across different lambda values. **(D)** Bar plot of the 14 features selected by LASSO regression and their respective coefficient weights in the final model.

For radiomics feature selection, we identified 22 ultrasonic radiomics features from 846 features through mutual information and significant differential expression (*P* < 0.05) filtering (**Figure 2A**). These selected radiomics features were combined with preoperative clinical features, yielding 36 total features for LASSO regression analysis (**Figure 2B**). This process selected 14 optimal features, including 10 clinical features and 4 radiomics features (**Figures 2C, D**). The distribution of these features across unilateral and bilateral disease groups is visualized in **Supplementary Figure 2**.

**Figure 3:**
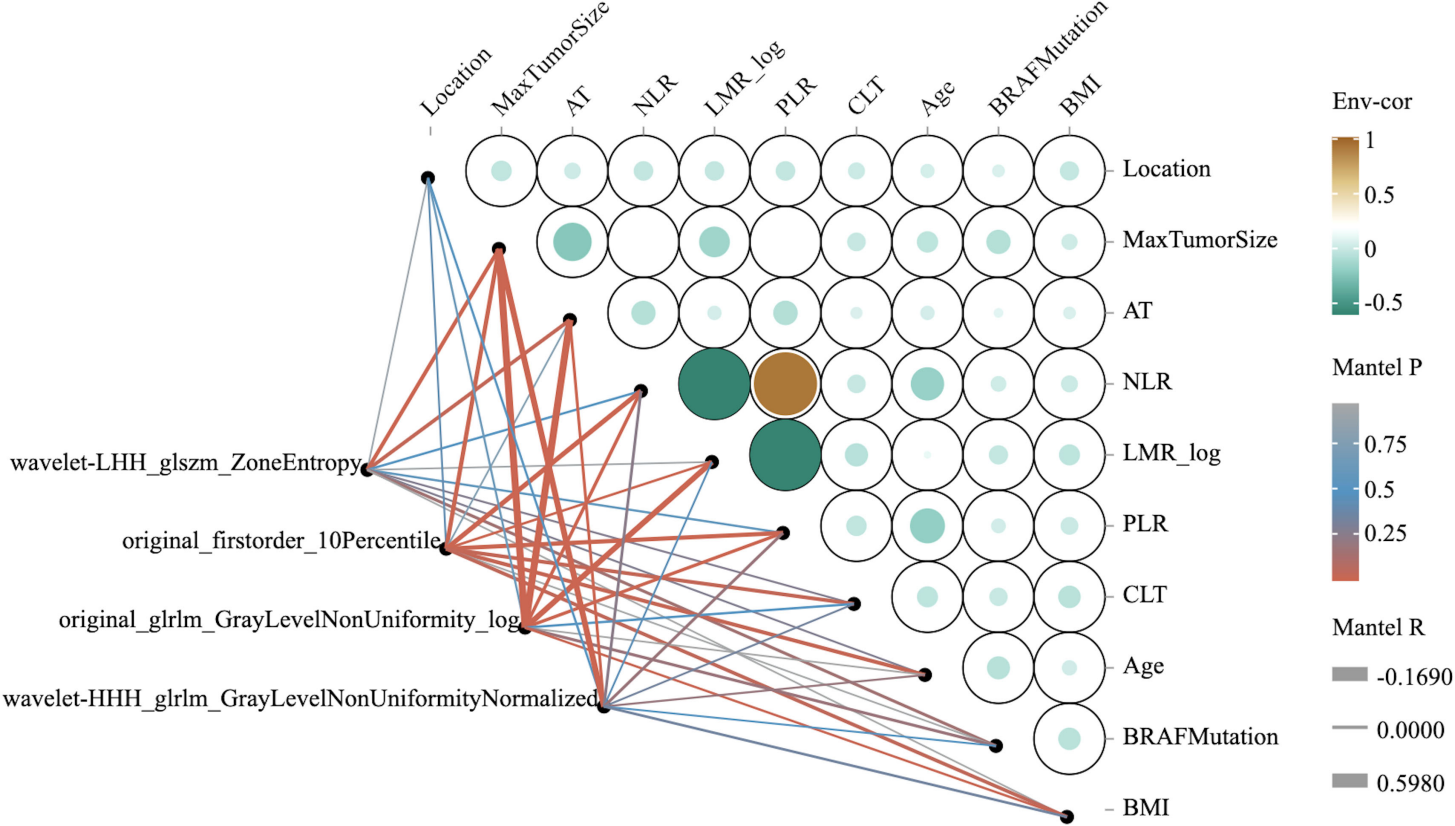
Correlation analysis between clinical and radiomics features. The circular correlation heatmap displays the strength and direction of associations between selected features. Circle size represents correlation magnitude, while color indicates direction (brown for positive, green for negative correlations). The network visualization (left) illustrates connections between radiomics features and clinical parameters with line thickness proportional to association strength.

The correlation analysis (**Figure 3**) revealed strong positive correlations between neutrophil-to-lymphocyte ratio (NLR) and platelet-to-lymphocyte ratio (PLR), while negative correlations were observed between lymphocyte-to-monocyte ratio (LMR) and both NLR and PLR. Radiomics features including wavelet-LHH_glszm_ZoneEntropy showed moderate correlations with tumor location and size parameters. Most clinical features exhibited weak to moderate correlations with each other, indicating their complementary value in the prediction models.

### Development and performance of prediction models

Multiple prediction models were developed for bilateral disease prediction. Model A employed logistic regression with preoperative and postoperative clinical parameters. Model B evaluated various machine learning algorithms using 14 LASSO-selected variables, with Support Vector Machine demonstrating superior performance achieving AUC of 0.724 (95% CI: 0.676-0.771) in training and 0.711 (95% CI: 0.606-0.812) in validation sets (**Supplementary Table 2**).

Model C compared different deep learning architectures including TresNet, ResNet variants, and Inception-3 for image-based prediction. TresNet achieved optimal performance with AUC of 0.894 (95% CI: 0.874-0.912) in training and 0.737 (95% CI: 0.637-0.829) in validation sets. Model D integrated Models B and C through logistic regression, achieving the highest performance with AUC of 0.970 (95% CI: 0.950-0.990) in training and 0.932 (95% CI: 0.900-0.960) in validation sets (**Supplementary Tables 2**, **3**).

In the training set, Model D demonstrated accuracy of 0.894, sensitivity of 0.844, and specificity of 0.994. Calibration plots revealed that Model D achieved optimal calibration in both datasets, with predicted probabilities closely matching observed frequencies compared to other models (**Supplementary Table 2**).

### External validation and clinical utility

**Figure 4:**
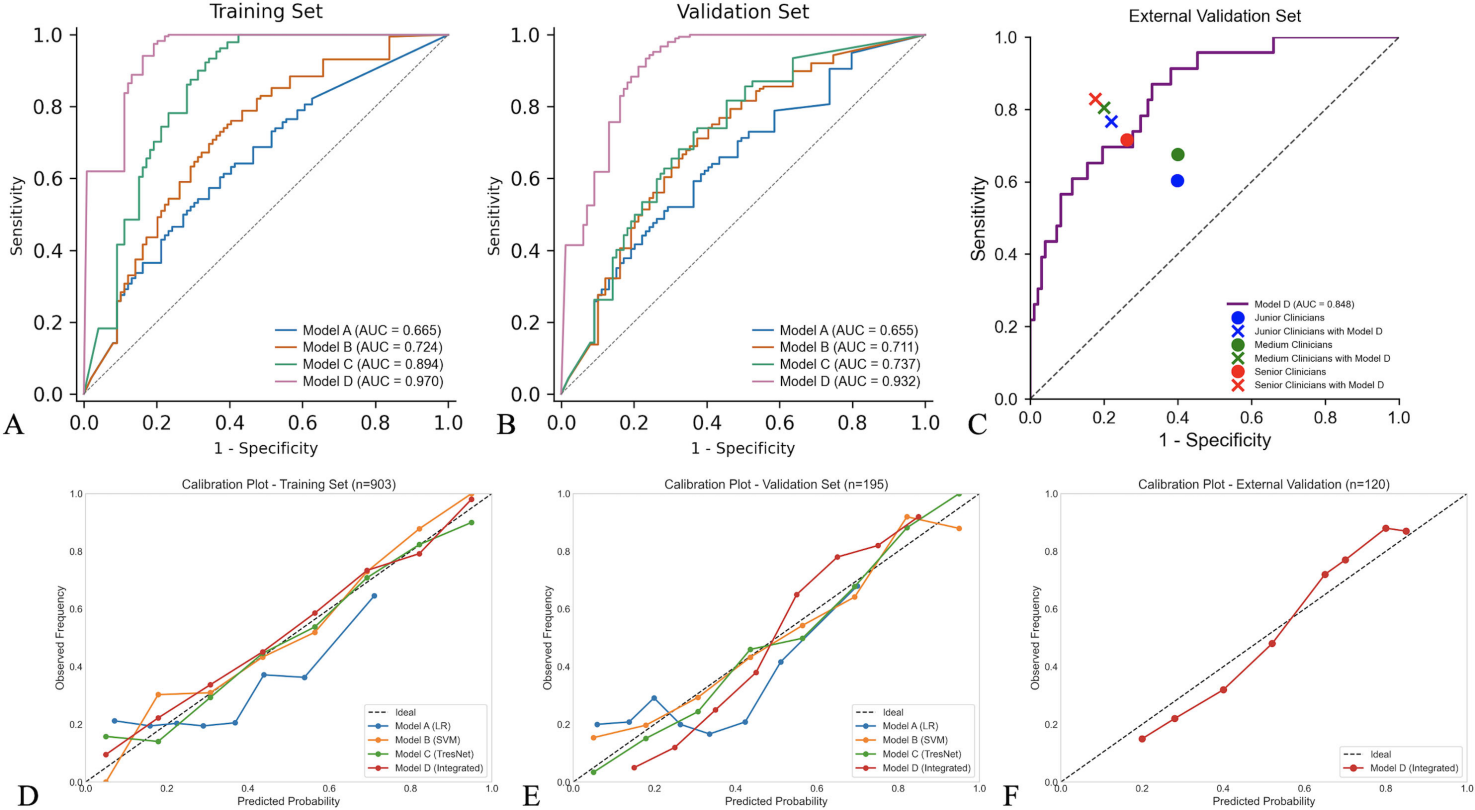
Performance evaluation of prediction models. **(A–C)** receiver operating characteristic curves for bilateral disease prediction in the training set, validation set, and external validation set. Points in panel **(C)** represent radiologist performance with and without Model D assistance. **(D–F)** Calibration plots comparing observed frequencies against predicted probabilities for all models across the three datasets.

In the external validation set (n=120), Model D maintained robust performance with an AUC of 0.848 (95% CI: 0.781-0.915), accuracy of 0.825, sensitivity of 0.792, and specificity of 0.867 (**Table 5**). The calibration plot (**Figure 4F**) confirmed Model D’s reliable probability estimations in the external validation cohort.

**Table 5:** Performance comparison of different models and radiologists in prediction of bilateral disease.

Modality	AUC	AUC 95% CI	ACC	Sensitivity	Specificity	PPV	NPV	F1 socre
Training set								
Model A (LR)	0.665	0.617-0.715	0.623	0.618	0.624	0.244	0.893	0.350
Model B (SVM)	0.724	0.676-0.771	0.687	0.660	0.692	0.296	0.912	0.409
Model C (TresNet)	0.894	0.874-0.912	0.799	0.858	0.770	0.651	0.916	0.740
Model D (Integrated)	0.970	0.950-0.990	0.894	0.844	0.994	0.970	0.950	0.890
Validation set								
Model A (LR)	0.655	0.547-0.746	0.605	0.583	0.609	0.226	0.882	0.326
Model B (SVM)	0.711	0.606-0.812	0.682	0.639	0.690	0.287	0.907	0.397
Model C (TresNet)	0.737	0.637-0.829	0.727	0.611	0.750	0.324	0.908	0.423
Model D (Integrated)	0.932	0.900-0.960	0.876	0.873	0.905	0.800	0.950	0.840
External validation set								
Model D (Integrated)	0.848	0.781-0.915	0.825	0.792	0.867	0.743	0.901	0.767
External validation set-before Model D (Integrated)								
Junior	N/A	N/A	0.583	0.592	0.574	0.521	0.635	N/A
Medium	N/A	N/A	0.655	0.662	0.648	0.592	0.722	N/A
Senior	N/A	N/A	0.720	0.717	0.723	0.675	0.765	N/A
External validation set-after Model D (Integrated)								
Junior	N/A	N/A	0.792	0.780	0.785	0.785	0.803	N/A
Medium	N/A	N/A	0.823	0.810	0.819	0.815	0.838	N/A
Senior	N/A	N/A	0.844	0.835	0.847	0.846	0.860	N/A

LR, Logistic regression; SVM, Support Vector Machine; AUC, area under the curve; CI, confidence interval; ACC, accuracy; PPV, positive predictive value; NPV, negative predictive value.

To assess clinical utility, we evaluated the diagnostic performance of radiologists with different experience levels before and after using Model D. Before implementation, the accuracy of junior, medium, and senior radiologists was 0.583, 0.655, and 0.720, respectively. After incorporating Model D, their accuracy significantly improved to 0.792, 0.823, and 0.844, respectively (**Table 5**; **Figure 4C**).

### Visualization analysis and model interpretation

**Figure 5:**
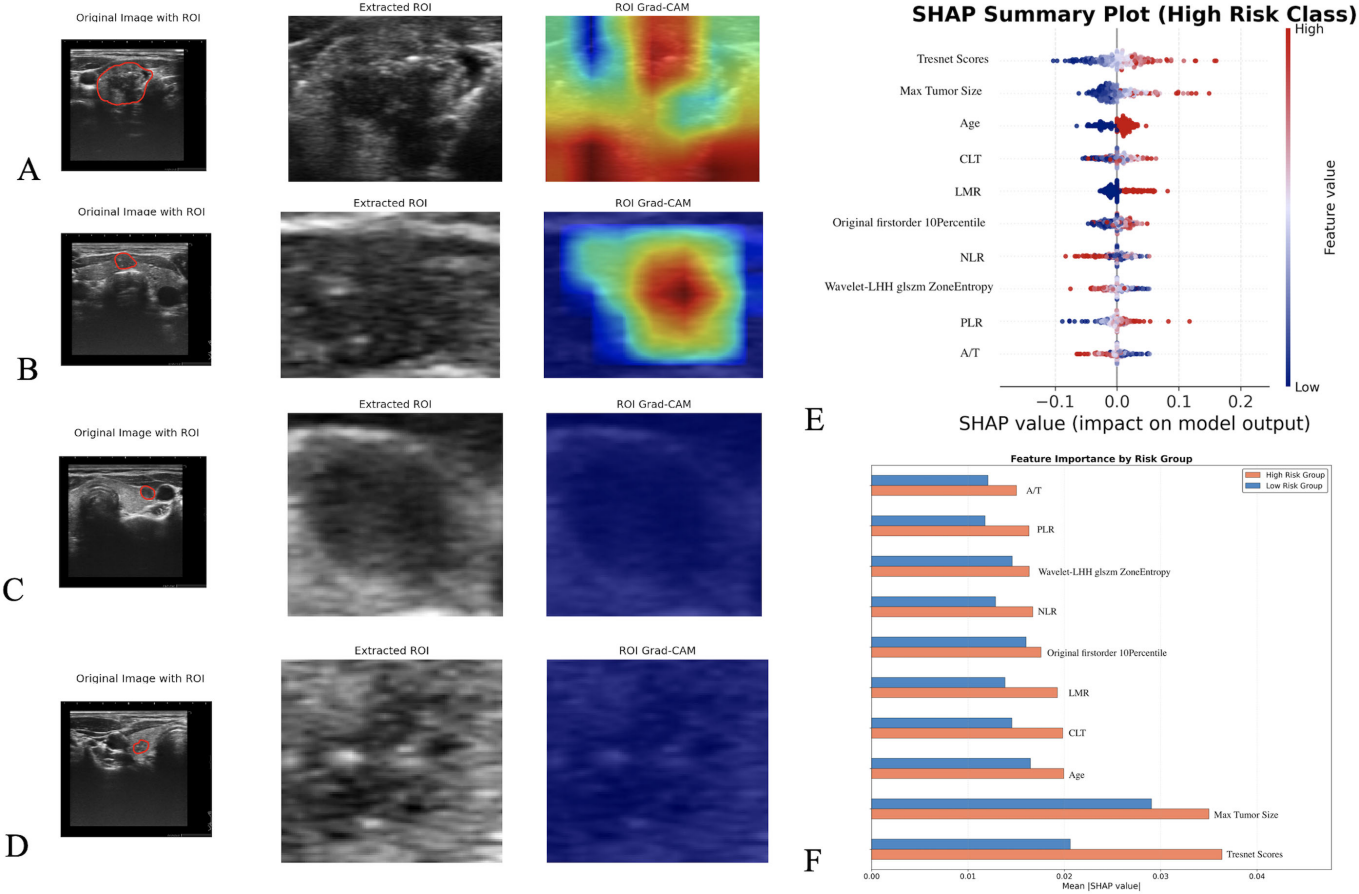
Model interpretability analysis using Grad-CAM and SHAP. Representative cases of bilateral disease prediction with positive **(A, B)** and negative **(C, D)** classification results. For each case, the original ultrasound image with ROI, region of interest outlined in red (left), the extracted ROI (middle), and the corresponding Grad-CAM heatmap (right) are shown. Red-yellow areas in positive cases indicate image regions strongly associated with bilateral disease prediction, while blue-green coloration in negative cases indicates features less predictive of bilateral disease. **(E)** SHAP summary plot showing feature impact on model output, with color indicating feature value (blue=low, red=high) and position showing SHAP value impact. **(F)** Mean absolute SHAP values by risk group, quantifying feature impact on predictions for high-risk (orange) and low-risk (blue) classifications.

Grad-CAM visualizations demonstrated distinct attention patterns between positive and negative cases (**Figure 5**). Positive cases showed high activation on irregular margins and heterogeneous echogenicity patterns with red-yellow heatmaps indicating features strongly associated with bilateral disease, while negative cases exhibited minimal activation with blue-green coloration representing less predictive features.

SHAP analysis of the integrated Model D identified key predictive features (**Figures 5E, F**). Tresnet scores and maximum tumor size demonstrated the strongest impact on predictions, with higher values associated with increased bilateral disease risk. Age showed significant influence, with older patients more frequently classified as high-risk. Laboratory parameters including CLT and LMR exhibited moderate effects, while radiomic features such as original firstorder 10Percentile and Wavelet-LHH glszm ZoneEntropy displayed distinctive patterns between risk groups.

**Figure 6:**
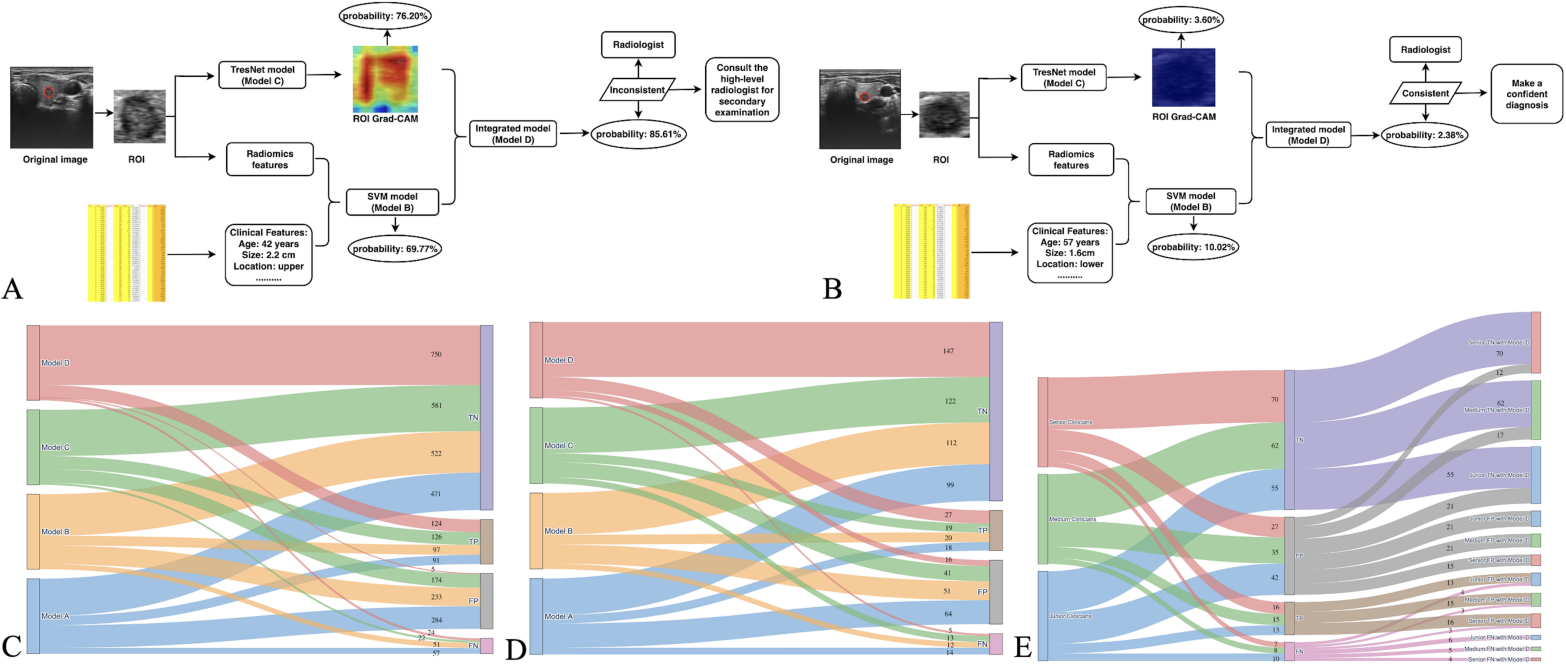
Clinical implementation and diagnostic flow analysis. **(A, B)** Workflow illustrations showing the integrated prediction approach with a positive case **(A)** (probability 85.61%) and negative case **(B)** (probability 2.38%). Each panel demonstrates how the deep learning model (Model C), radiomics-based model (Model B), and integrated model (Model D) contribute to the final prediction. **(C–E)** Sankey diagrams illustrating prediction flow in the training set **(C)**, validation set **(D)**, and external validation set before and after Model D implementation **(E)**. Width of flow bands indicates case volume, with diagnostic accuracy improvements across all radiologist experience levels after model integration.

Clinical application workflow demonstrated the integrated model’s effectiveness in external validation (**Figures 6A, B**). A positive case showed Model C (76.20%) and Model B (69.77%) detecting suspicious features, with Model D synthesizing these into higher confidence prediction (85.61%). A negative case exhibited consistently low probabilities across all models (3.60%, 10.02%, and 2.38%), providing clear diagnostic guidance.

Sankey diagrams illustrated decision flow and predictive contributions across datasets (**Figures 6C–E**). Model D achieved superior performance with 750 true negatives and 126 true positives in training, and 147 true negatives and 19 true positives in validation. Implementation of Model D significantly improved diagnostic accuracy across experience levels, with junior radiologists showing the greatest improvement from 58.3% to 79.2% (+20.9%), effectively reducing performance gaps between different experience levels.

## Discussion

In this comprehensive study, we investigated the clinicopathological characteristics, recurrence patterns, and predictive factors of bilateral disease in patients with the classic subtype of PTC. Our findings provide valuable insights that may significantly improve the understanding and management of multifocal PTC.

The prevalence of multifocality in our cohort was 26.1%, which aligns with previous reports ranging from 20% to 36.1% (2, 3). Among multifocal cases, bilateral disease represented a substantial proportion (62.7%), underscoring the critical importance of accurate preoperative identification of these patients. Our results demonstrated that bilateral disease was associated with several adverse clinicopathological features, including larger tumor size, higher incidence of central and lateral lymph node metastasis, and increased frequency of isthmus location compared to both unifocal disease and unilateral multifocality. These findings corroborate previous studies by Rodriguez Schaap et al. (3) and Li et al. (19), which reported similar aggressive characteristics in bilateral PTC.

It is noteworthy that our survival analysis revealed bilateral disease, rather than simply the number of tumor foci, was significantly associated with worse recurrence-free survival. This observation is particularly important because it challenges the conventional view that multifocality by itself a risk factor for recurrence. Instead, our results suggest that the spatial distribution of tumor foci (bilateral versus unilateral) has greater prognostic relevance than the mere number of lesions. This finding aligns with previous studies that bilaterality as an independent risk factor for persistent and recurrent disease in PTCs (6, 7, 19). Furthermore, Harries et al. (20) reported that multifocality alone was not an indication for completion thyroidectomy in PTC, supporting our observation that unilateral multifocality does not significantly increase recurrence risk compared to unifocal disease.

Our multivariate analysis identified several independent predictors of bilateral lesions, including BRAF V600E mutation, A/T ratio ≥1, high-volume central lymph node metastasis, and the size of metastatic lymph nodes in the central compartment. The strong association between BRAF V600E mutation and bilateral lesions in our cohort is particularly noteworthy, consistent with findings by Fang et al. (21), who reported that up to 84.0% of multifocal PTC lesions carried the BRAF V600E mutation. This significant correlation suggests an important role of BRAF V600E in the occurrence of bilateral PTC. However, despite BRAF V600E being an important predictor, the biological behavior of bilateral lesions may be influenced by more complex molecular mechanisms. Fang et al. (21) found that in multifocal PTC, 64.0% of cases had the same mutation spectrum in both lesions, indicating intrathyroidal metastasis, while 18.0% showed completely different mutation patterns, suggesting independent origins. Notably, bilateral lesions were more likely to originate from independent origins, implying multiple primary clones evolving separately (22). This tumor heterogeneity increases treatment difficulty and recurrence possibility, thereby leading to a higher risk of recurrence.

The significant innovation of our study lies in the development and validation of a multimodal prediction model that integrates preoperative clinical parameters, inflammatory markers, radiomics features, and ultrasound images for the preoperative identification of bilateral lesions. Network visualization demonstrated specific associations between clinical features and radiomics characteristics: NLR significantly correlated with wavelet-LHH_glszm_ZoneEntropy, suggesting inflammatory status may be associated with tumor texture features; BRAF mutation correlated with original_glrlm_GrayLevelNonUniformity_log, indicating genetic alterations may be reflected in image gray-level non-uniformity (23); maximum tumor size connected with multiple wavelet transformation features related to non-uniformity, consistent with larger tumors exhibiting greater tissue heterogeneity. Most clinical features exhibited weak to moderate correlations, suggesting their complementary value in predictive modeling.

This multimodal prediction model (Model D) employed an ensemble approach that integrated the strengths of logistic regression-based clinical and ultrasound features, radiomics, and deep learning models, forming a complementary diagnostic framework: logistic regression ensured basic interpretability and clinical relevance, radiomics captured highly specific morphological features, while deep learning extracted complex hierarchical patterns, achieving high-sensitivity identification (24, 25). Compared to individual component models, the superior performance of our multimodal model demonstrated the complementary value of different predictive approaches, an innovation particularly important given that conventional ultrasound shows low sensitivity (61%) and specificity (31%) in detecting multifocality and bilaterality (26).

This multimodal integration not only enhanced predictive performance but also improved model user-friendliness for clinicians. SHAP feature attribution analysis revealed that TresNet deep learning score constituted the largest contribution proportion within the multimodal model. TresNet extracts high-dimensional features directly from raw ultrasound images, overcoming operator dependency, while capturing complex features including tumor location, texture, and microcalcifications, and identifying specific patterns associated with bilateral disease, processing non-linear relationships in high-dimensional data more effectively than handcrafted radiomics features, thus achieving higher sensitivity (27, 28). Additionally, the implementation of advanced visualization technique Grad-CAM identified specific image regions and features that contributed most significantly to bilateral lesion prediction, substantially enhancing model interpretability (29). Our clinical utility assessment indicated that the integrated model significantly reduced false-positive and false-negative results across all experience levels, with junior radiologists benefiting most substantially. Notably, the model had the most significant impact on narrowing the performance gap between junior and senior radiologists, effectively reducing experience-dependent variations in diagnostic accuracy and creating more standardized assessment capabilities.

Our study has several limitations. First, its retrospective nature introduces potential selection bias. Second, the relatively short follow-up period (median 29.9 months) may not capture all recurrence events, particularly late recurrences. Third, while our model showed robust performance in external validation, further multicenter studies with larger and more diverse cohorts are needed to establish its generalizability. Fourth, our study focused primarily on classic PTC and may not be applicable to other PTC variants or thyroid cancer subtypes. Finally, molecular profiles beyond BRAF V600E mutation were not comprehensively assessed, potentially missing additional biological insights. Additionally, detailed metabolic data beyond BMI — such as lipid profiles, glycemic indices, and adipokine levels — were not systematically collected in this retrospective cohort, limiting further exploration of the metabolic–multifocality relationship in PTC. Future prospective studies incorporating comprehensive molecular profiling and longer follow-up periods may further refine risk stratification and personalized treatment approaches for patients with multifocal PTC.

## Conclusions

In conclusion, our findings suggest that bilateral disease, rather than multifocality alone, is a significant risk factor for recurrence in PTC. Through the integration of diverse data modalities—including clinical parameters, inflammatory biomarkers, quantitative radiomics signatures, and high-resolution ultrasound images—our artificial intelligence-driven multimodal model demonstrated promising performance in preoperatively identifying bilateral disease, potentially guiding surgical planning and postoperative management.

## Data Availability

The raw data supporting the conclusions of this article will be made available by the authors, without undue reservation.

## Supplementary material

Supplementary Figure 1 Study flowchart. Diagram illustrating patient selection and cohort stratification. Of 1,098 enrolled PTC patients, 811 presented with unifocal disease and 287 with multifocal disease. The multifocal group included 107 unilateral multifocal PTCs and 180 bilateral PTCs. Within bilateral cases, 32 had contralateral occult PTC. The training set (n=903) comprised 148 non-incidentally discovered bilateral cases and 755 unilateral cases, while the validation set (n=195) included 32 incidentally discovered bilateral cases and 163 unilateral cases. An external validation set (n=120) was used for final model assessment.

Supplementary Figure 2 Feature distribution by multifocality groups. Comparative analysis of clinical and radiomics features between unilateral (blue) and bilateral (green) disease patterns. Significant differences were observed in tumor location, maximum tumor size, A/T ratio, inflammatory markers (NLR, LMR, PLR), radiomics features, CLT prevalence, BRAF mutation status, age, and BMI between groups. Box plots display median, interquartile range, and distribution of continuous variables, while bar charts present percentages for categorical variables.

Supplementary Table 1 Characteristics of patients with contralateral occult PTC in bilateral disease.

Supplementary Table 2 Performance of the different models in training and validation set.

Supplementary Table 3 Structure of the TresNet used in the paper.

## Glossary

PTC: Papillary Thyroid Carcinoma
AI: Artificial Intelligence
AUC: Area Under the Receiver Operating Characteristic Curve
LASSO: Least Absolute Shrinkage and Selection Operator
BMI: Body Mass Index
CLT: Chronic Lymphocytic Thyroiditis
DICOM: Digital Imaging and Communications in Medicine
ROI: Region of Interest
RFS: Recurrence-Free Survival
CI: Confidence Interval
OR: Odds Ratio
HR: Hazard Ratio
ROC: Receiver Operating Characteristic
PPV: Positive Predictive Value
NPV: Negative Predictive Value
Grad-CAM: Gradient-weighted Class Activation Mapping
SHAP: SHapley Additive exPlanations
UFD: Unifocal Disease
MFD: Multifocal Disease
A/T: Aspect to Transverse
CLNM: Central Lymph Node Metastasis
LN: Lymph Node
NLR: Neutrophil-to-Lymphocyte Ratio
PLR: Platelet-to-Lymphocyte Ratio
LMR: Lymphocyte-to-Monocyte Ratio
SVM: Support Vector Machine.
