# Supplementary material for: Bilateral disease in the classic subtype of papillary thyroid carcinoma: clinical significance and development of an artificial intelligence-based multimodal prediction model
Source: Front Endocrinol (Lausanne). 2026 May 20;17:1759451. doi: 10.3389/fendo.2026.1759451 (PMC13229819; doi:10.3389/fendo.2026.1759451)
Supplement: Supplementary Figure 1 — Study flowchart. Diagram illustrating patient selection and cohort stratification. Of 1,098 enrolled PTC patients, 811 presented with unifocal disease and 287 with multifocal disease. The multifocal group included 107 unilateral multifocal PTCs and 180 bilateral PTCs. Within bilateral cases, 32 had contralateral occult PTC. The training set (n=903) comprised 148 non-incidentally discovered bilateral cases and 755 unilateral cases, while the validation set (n=195) included 32 incidentally discovered bilateral cases and 163 unilateral cases. An external validation set (n=120) was used for final model assessment. [file DataSheet1.docx]

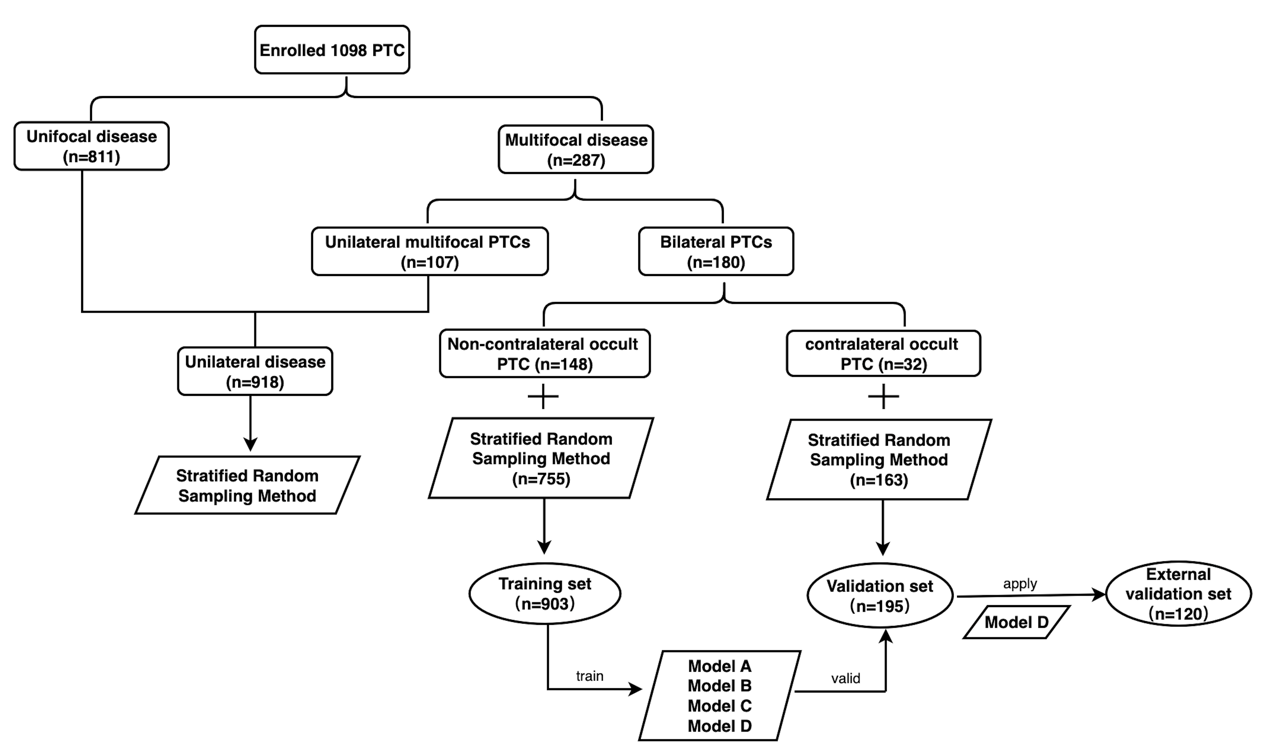


Supplementary Figure 1. Study flowchart. Diagram illustrating patient selection and cohort stratification. Of 1,098 enrolled PTC patients, 811 presented with unifocal disease and 287 with multifocal disease. The multifocal group included 107 unilateral multifocal PTCs and 180 bilateral PTCs. Within bilateral cases, 32 had contralateral occult PTC. The training set (n=903) comprised 148 non-incidentally discovered bilateral cases and 755 unilateral cases, while the validation set (n=195) included 32 incidentally discovered bilateral cases and 163 unilateral cases. An external validation set (n=120) was used for final model assessment.


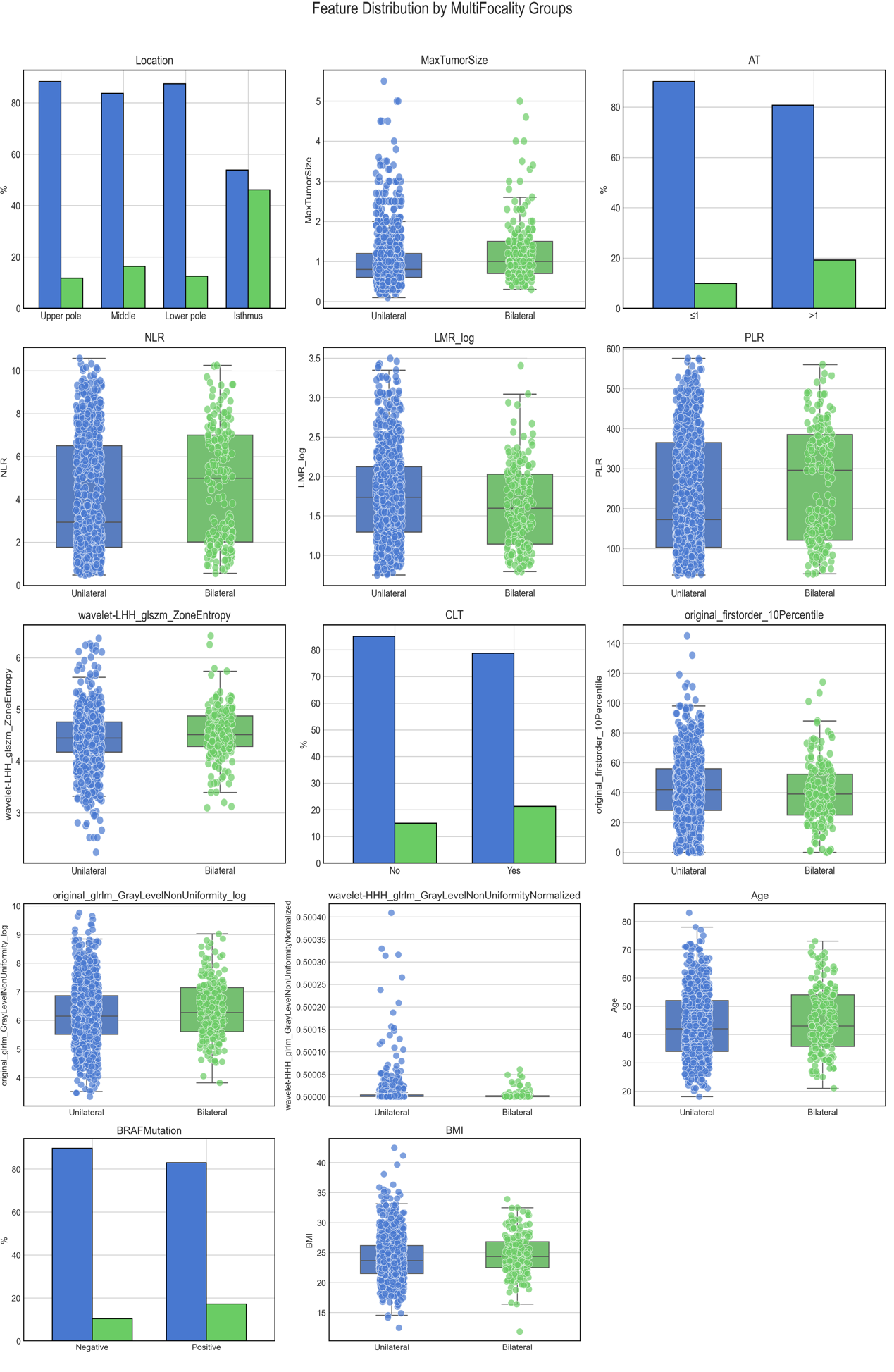


Supplementary Figure 2. Feature distribution by multifocality groups. Comparative analysis of clinical and radiomics features between unilateral (blue) and bilateral (green) disease patterns. Significant differences were observed in tumor location, maximum tumor size, A/T ratio, inflammatory markers (NLR, LMR, PLR), radiomics features, CLT prevalence, BRAF mutation status, age, and BMI between groups. Box plots display median, interquartile range, and distribution of continuous variables, while bar charts present percentages for categorical variables.

Supplementary Table 1. Characteristics of patients with contralateral occult PTC in bilateral disease

| Total no. of patients | 32 |
| --- | --- |
| Sex (Female) | 21 (65.6%) |
| Age ( <55 years) | 24 (75.0%) |
| Contralateral tumor size, cm | 0.71 ± 0.44 |
| TIRADS for contralateral occult PTC |  |
| 1 or 2 | 0 (0.0%) |
| 3 | 10 (31.3%) |
| 4 | 9 (28.1%) |
| 5 | 4 (12.5%) |
| N/a | 9 (28.1%) |
| Preop FNA performed for contralateral nodule (Yes) | 6 (18.8%) |
| Surgery methods |  |
| TT + bilateral CND | 21 (65.6%) |
| Near-TT/TT + Unilateral CND | 6 (18.8%) |
| TT + mRND | 5 (15.6%) |
| Risk stratification |  |
| Low | 17 (53.1%) |
| Intermediate | 15 (46.9%) |
| High | 0 (0.0%) |
| Recurrence (Presence) | 1 (3.1%) |

*TIRADS* thyroid imaging, reporting and data system, *FNA* fine-needle aspiration, *CND* central neck dissection, *TT* total thyroidectomy, *mRND* modified radical neck dissection.

Supplementary Table 2. Performance of the different models in training and validation set

| Models | AUC | AUC 95% CI | ACC | Sensitivity | Specificity | PPV | NPV | Kappa | F1 Score | Loss |
| --- | --- | --- | --- | --- | --- | --- | --- | --- | --- | --- |
| **Model B-Training set** | | |  |  |  |  |  |  |  |  |
| RF | 0.705 | 0.652-0.758 | 0.675 | 0.645 | 0.680 | 0.282 | 0.905 | 0.388 | 0.394 | 0.240 |
| GBM | 0.712 | 0.661-0.763 | 0.680 | 0.650 | 0.685 | 0.290 | 0.908 | 0.395 | 0.400 | 0.228 |
| **SVM** | **0.724** | **0.676-0.771** | **0.687** | **0.660** | **0.692** | **0.296** | **0.912** | **0.401** | **0.409** | **0.210** |
| XGB | 0.718 | 0.669-0.767 | 0.683 | 0.655 | 0.688 | 0.295 | 0.910 | 0.399 | 0.405 | 0.215 |
| KNN | 0.690 | 0.640-0.740 | 0.662 | 0.630 | 0.670 | 0.270 | 0.900 | 0.375 | 0.380 | 0.260 |
| **Model B-Validation set** | | |  |  |  |  |  |  |  |  |
| RF | 0.692 | 0.585-0.795 | 0.667 | 0.625 | 0.675 | 0.275 | 0.890 | 0.378 | 0.380 | 0.265 |
| GBM | 0.702 | 0.598-0.805 | 0.672 | 0.630 | 0.682 | 0.280 | 0.895 | 0.385 | 0.387 | 0.252 |
| **SVM** | **0.711** | **0.606-0.812** | **0.682** | **0.639** | **0.690** | **0.287** | **0.907** | **0.390** | **0.397** | **0.235** |
| XGB | 0.706 | 0.600-0.810 | 0.678 | 0.635 | 0.686 | 0.283 | 0.902 | 0.388 | 0.392 | 0.240 |
| KNN | 0.682 | 0.580-0.780 | 0.658 | 0.618 | 0.667 | 0.270 | 0.885 | 0.370 | 0.375 | 0.278 |
| **Model C-Training set** | | |  |  |  |  |  |  |  |  |
| **TresNet** | **0.894** | **0.874-0.912** | **0.799** | **0.858** | **0.770** | **0.651** | **0.916** | **0.726** | **0.740** | **0.127** |
| ResNet 34 | 0.846 | 0.820-0.872 | 0.768 | 0.812 | 0.742 | 0.602 | 0.893 | 0.684 | 0.691 | 0.165 |
| ResNet 101 | 0.852 | 0.827-0.878 | 0.775 | 0.820 | 0.750 | 0.612 | 0.897 | 0.692 | 0.701 | 0.158 |
| ResNet 18 | 0.835 | 0.808-0.862 | 0.760 | 0.800 | 0.735 | 0.590 | 0.886 | 0.675 | 0.680 | 0.175 |
| Inception 3 | 0.830 | 0.802-0.858 | 0.752 | 0.792 | 0.728 | 0.580 | 0.881 | 0.668 | 0.670 | 0.185 |
| **Model C-Validation set** | | |  |  |  |  |  |  |  |  |
| **TresNet** | **0.737** | **0.637-0.829** | **0.727** | **0.611** | **0.750** | **0.324** | **0.908** | **0.412** | **0.423** | **0.205** |
| ResNet 34 | 0.706 | 0.605-0.795 | 0.702 | 0.590 | 0.728 | 0.308 | 0.895 | 0.388 | 0.401 | 0.228 |
| ResNet 101 | 0.712 | 0.610-0.802 | 0.712 | 0.598 | 0.735 | 0.312 | 0.900 | 0.392 | 0.410 | 0.222 |
| ResNet 18 | 0.698 | 0.596-0.785 | 0.692 | 0.582 | 0.720 | 0.302 | 0.890 | 0.380 | 0.395 | 0.235 |
| Inception 3 | 0.690 | 0.590-0.775 | 0.685 | 0.575 | 0.715 | 0.298 | 0.882 | 0.375 | 0.390 | 0.242 |

*RF* Random Forest*, GBM* Gradient Boosting Machine, *SVM* Support Vector Machine,

*XGB* eXtreme Gradient Boosting*, KNN* K-Nearest Neighbors*, AUC* area under the curve, *CI* confidence interval, *ACC* accuracy, *PPV* positive predictive value, *NPV* negative predictive value

Supplementary Table 3**.** Structure of the TresNet used in the paper

| Name | Output Size | Layer | Parameter Setting | Number of Blocks |
| --- | --- | --- | --- | --- |
| Input Layer | 224 × 224 | Input Image | Grayscale, resized to 224 × 224 pixels | 1 |
| Root | 112 × 112 | Conv + Space-to-Depth | k7, c64, s2, p3 | 1 |
|  | 112 × 112 | Anti-Aliased Max Pooling | k3, s2, p0, d1 | 1 |
| Block 1 | 112 × 112 | Residual Block + SE Module | k3, c64, s1, SE included | 3 |
| Block 2 | 56 × 56 | Residual Block + SE Module | k3, c128, s2, SE included | 4 |
| Block 3 | 28 × 28 | Residual Block + SE Module | k3, c256, s2, SE included | 6 |
| Block 4 | 14 × 14 | Residual Block + SE Module | k3, c512, s2, SE included | 3 |
| Head Layer | 1 × 1 | Global Average Pooling + Fully Connected | Output = 2 (binary classification) | 1 |

k: Kernel size; c: Number of output channels; s: Stride of the convolution or pooling operation; p: Padding applied to the input tensor; d: Dilation rate in dilated convolutions; SE: Squeeze-and-Excitation module for channel-wise feature recalibration; Residual Block: A block with skip connections to ease gradient flow and improve training; Global Average Pooling: A pooling operation averaging each feature map; Fully Connected: A dense layer for classification.
